# Diagnosis of Early Cervical Cancer with a Multimodal Magnetic Resonance Image under the Artificial Intelligence Algorithm

**DOI:** 10.1155/2022/6495309

**Published:** 2022-03-23

**Authors:** Zhenge Zhang, Chongyuan Zhang, Li Xiao, Shuirong Zhang

**Affiliations:** Department of Gynecology Ward 2, Jingzhou Central Hospital, Jingzhou Hospital, Yangtze University, Jingzhou 434020, Hubei, China

## Abstract

This research was conducted to explore the value of multimodal magnetic resonance imaging (MRI) based on the alternating direction algorithm in the diagnosis of early cervical cancer. 64 patients diagnosed with early cervical cancer clinicopathologically were included, and according to the examination methods, they were divided into A group with conventional multimodal MRI examination and B group with the multimodal MRI examination under the alternating direction algorithm. The diagnostic results of two types of multimodal MRI for early cervical cancer staging were compared with the results of clinicopathological examination to judge the application value in the early diagnosis of cervical cancer. The results showed that in the 6 randomly selected samples of early cervical cancer patients, the peak signal-to-noise ratio (PSNR) and structural similarity image measurement (SSIM) of multimodal MRI images under the alternating direction algorithm were significantly higher than those of conventional multimodal MRI images and the image reconstruction was clearer under this algorithm. By comparing MRI multimodal staging, statistical analysis showed that the staging accuracy of B group was 75%, while that of A group was only 59.38%. For the results of postoperative medical examinations, the examination consistency of B group was better than that of A group, with a statistically significant difference (*P* < 0.05). The area under the receiver operating characteristic (ROC) curve (AUC) of B group was larger than that of A group; thus, sensitivity was improved and misdiagnosis was reduced significantly. Multimodal MRI under the alternating direction algorithm was superior to conventional multimodal MRI examination in the diagnosis of early cervical cancer, as the lesions were displayed more clearly, which was conducive to the detection rate of small lesions and the staging accuracy. Therefore, it could be used as an ideal MRI method for the assistant diagnosis of cervical cancer staging.

## 1. Introduction

Cervical cancer is a malignant tumor with high incidence among gynecological diseases. It is one of the four most common cancers and poses a serious threat to women's life and health [1]. Epidemiological investigations have found that cervical cancer and its carcinoma in situ are closely related to factors such as sexually transmitted diseases, smoking, and premature sex (<16 years old). In addition, the diseased population is becoming younger and younger [2, 3]. Generally, cervical cancer has no significant feature in the early stage and occasionally manifests as increased vaginal discharge. The clinical methods for early cervical cancer diagnosis are the three examinations of colposcopy, cervical cytology, and cervical biopsy [4, 5]. However, biopsy is invasive and tissue sampling determines its accuracy. Early lesions in tumor tissues are not easy to find and occasionally appear as punctate lesions, which may lead to missed diagnosis. Therefore, an accurate and less traumatic diagnostic method is needed. The accuracy of early diagnosis and clinical staging of cervical cancer is of great significance for improving the prognosis and survival rate of cervical cancer patients, and overestimating or underestimating the severity of cancer tissue lesions will make an adverse effect on the prognosis [6].

Clinically, the initial diagnosis of cervical cancer is generally a gynecological examination, which is less reliable in the diagnosis of parauterine infiltration, degree of infiltration, tumor size, and the distant metastases of lymph nodes or pelvic cavity [7]. At present, clinical imaging methods, such as magnetic resonance imaging (MRI) plain scan, ultrasound, and computer tomography (CT), are used to evaluate the staging of cervical cancer [8, 9]. Ultrasound is simple, fast, and easy to operate, with a relatively cheap price. However, the anatomical scope of ultrasound is limited and the clarity of the tissue structure and lesions detected is much lower than that of CT and MRI [10]. CT can give a high density resolution, which can detect soft tissue structures or organs with small density differences accurately and can measure quantified CT parameter values, but it still cannot accurately observe the distant metastasis, pelvic infiltration, and the situation of rectum and bladder for cervical cancer [11]. MRI examination has the advantages of high tissue resolution and multisequence and multiparameter imaging, which are advantageous in the diagnosis and staging of cervical cancer. Currently, the monomodal, static, and planar MRI with morphological imaging has been developed to be three dimensional and dynamically multimodal with functional imaging [12]. The multimodal imaging summarizes the medical images under different modal imaging methods, so as to make up for deficiencies of each method; thereby, more effective data can be obtained, and finally, a clearer display of the lesions and efficient diagnosis can be realized. It combines functional imaging and anatomical imaging, so that the structural and functional information of the tissue are reflected, which is more conducive to the diagnosis of diseases [13].

However, compared with other medical imaging technologies, multimodal MRI also has some shortcomings and limitations. For example, the patient needs to burden high medical expenses, the scanning time is relatively longer, and the imaging resolution is low for it is easily affected by scanning time, signal-to-noise ratio, or more factors [14]. With the continuous development of computer intelligent algorithms, a new medical imaging technology called partial parallel imaging has emerged in the medical field. As an alternating direction algorithm, it is mainly applied and collects Fourier components concurrently in each receiver, as multiple receivers surround the scanned object, thereby the image quality and reconstruction speed are improved [15]. Without further processing, artifacts will appear in the final image, which will lead to the deteriorated image quality. For a higher-quality reconstructed image, a fast optimization algorithm is required to reconstruct the image.

Therefore, in this study, the alternating direction algorithm was applied in this study, as it has been used in the partial parallel imaging. Meanwhile, the algorithm was combined with multimodal MRI to diagnose patients with early cervical cancer. With the clinical pathological examination results, its application effect in diagnosis of early cervical cancer was evaluated.

## 2. Method

### 2.1. Research Subjects

In this study, 64 patients with early cervical cancer were selected in hospital from May 2018 to March 2021. They were 21–57 years old, with a median age of 43.8 years old. All cases were confirmed by postoperative pathological examinations. According to the examination methods, they were divided into two groups. In A group, the patients received conventional multimodal MRI examination, while in B group, they underwent multimodal MRI examination based on the alternating direction algorithm. The patients included in this study signed the informed consent forms, and the research process had been approved by the ethics committee of the hospital.

The patients in this study met the following inclusion criteria. They had no contraindications to MRI and were newly diagnosed with cervical cancer. Their MRI images are good in the quality without obvious distortion or artifacts. They could cooperate to complete the examinations and participate in the study voluntarily. With the exclusion criteria, patients had not undergone surgery without complete pathological data were excluded. Those who did not have complete data of MRI examination, those who have pelvic lymph node metastasis, and those with cervical cancer of stage II-B or above were also excluded.

### 2.2. Staging Standards of Cervical Cancer

A 3.0T superconducting magnetic resonance imaging system was used. All serial images of MRI were read blindly and staged by two senior doctors and a postgraduate imaging student. The images were analyzed and measured; if there was any divergence in the staging, it was determined after discussion. The staging standards of International Federation of Gynecology and Obstetrics (FIGO) [16] are shown in Table 1.

### 2.3. Alternating Direction Algorithm

In this study, the image reconstruction method based on image domain and coil sensitivity coding was applied, as shown below:

(1) $$\begin{array}{c} {\text{MFS}}_{j}u=f_{j}. \end{array}$$

According to the equation above, some *k* spatial data *f*_*j*_ obtained by the *j*-th receiver and the sensitivity mapping *S*_*j*_ were correlated with the mask *M*. *F* was the Fourier transform, *f*_*j*_ was the number of receivers, and *S* was the sensitivity mapping, which represented the vector of Fourier coefficients of the receiver.

According to (1), the reconstruction equation of image *u* was worked out by solving the least squares. The reconstruction equation was expressed as follows: .

(2) $$\begin{array}{c} \underset{u\in C^{N}}{\min}\sum_{j=1}^{k}{\text{MFS}}_{j}u-f_{j2}^{2}. \end{array}$$

In the above equation, ‖·‖_2_^2^ was 2-norm (also called Euclidean norm), and *k* was the number of channels (or receivers). Since the matrix obtained by *MFS*_*j*_ was ill-conditioned in (2), the minimization might be ill-conditioned as well. To reduce the influence of ill-condition, the recent SENSE model had been added into the energy functional function through a regularization term. Finally, the basic sparsity of the MR image in the finite difference region was utilized to optimize the rapid reconstruction of the image, by solving the optimization issue of the following equation:

(3) $$\begin{array}{c} \underset{u\in C^{N}}{\text{min}}u_{TV}+\lambda \sum_{j=1}^{K}\left\|{\text{MFS}}_{j}u-f_{j2}^{2}\right\|. \end{array}$$

In (3), ‖·‖_*TV*_ was the total variational norm, and *λ* > 0 was the parameter corresponding to the relative weight of the data fidelity term. Then, (4) was worked out.

(4) $$\begin{array}{c} \overset{K}{\underset{j=1}{\sum }}\left\|{\text{MFS}}_{j}u-f_{j2}^{2}\right\|. \end{array}$$

In (3), ‖·‖_*TV*_ controlled the solution, and the general form of the image reconstruction was expressed as follows:

(5) $$\begin{array}{c} \underset{u\in C^{N}}{\min}Y\left(u\right)+H\left(u\right), \end{array}$$

where *Y* represented a convex function, perhaps a non-differentiable function, *H* was a convex function that was continuously differentiable. In the image reconstruction based on the total variational model, *Y* and *H* had the following form as shown below:

(6) $$\begin{array}{c} Y\left(u\right)={\left\|u\right\|}_{TV},H\left(u\right)=\lambda {\left\|A_{tt}\right\|}^{2}f, \end{array}$$

*f* was the measured data; and *A* was the matrix that described the imaging equipment or data acquisition mode, which might be complex and ill-conditioned. The matrix *A* in parallel MRI was expressed as below:.

(7) $$\begin{array}{c} \text{A}=\left(\begin{array}{c} {\text{MFS}}_{1}\\ \vdots \\ {\text{MFS}}_{K} \end{array}\right). \end{array}$$

To solve the lack of smoothness of *Y* in (6), an auxiliary variable *v* was added to obtain the equivalent constraint issue, which was expressed as follows: .

(8) $$\begin{array}{c} \underset{u\in C^{N}}{\min}Y\left(v\right)+H\left(u\right),\text{s}.\text{t}u=v,u,v\in C^{N}. \end{array}$$

After that, the equivalent constraint issue was transformed into an unconstrained issue through the second penalty, as shown in the following equation:.

(9) $$\begin{array}{c} \underset{u,v}{\min}Y\left(v\right)+H\left(u\right)+\alpha {\left\|v-u\right\|}_{2}^{2}. \end{array}$$

In (9), *α* was a parameter, and an auxiliary variable *v* was introduced, so that the smooth term *H* and the nondifferentiable term *Y* could be handled independently to a certain extent. At this time, the penalty term issue was dealt with preferentially: the minimized *v* was found first by fixing *u*, and then the minimized *u* was obtained by fixing *v*, which are shown in the following equation:

(10) $$\begin{array}{c} v^{k+1}=\arg\underset{v}{\min}{\left\|v\right\|}_{\text{TV}}+\alpha {\left\|v-u\right\|}_{2}^{2},\\ u^{k+1}=\arg\underset{u}{\min}{\left\|\text{Au}-f\right\|}_{2}^{2}+\alpha {\left\|v-u\right\|}_{2}^{2}, \end{array}$$

*v*^*k*+1^ in the (10) represented the first sub-issue under the total variation, and *u*^*k*+1^ represented the second sub-issue. The primal-dual mixed gradient (PDHG) algorithm could be introduced to solve the first sub-issue, then the *v*^*k*+1^ in (10) could be expressed as given below:

(11) $$\begin{array}{c} \underset{v}{\min}\overset{N}{\underset{i=1}{\sum }}{\left\|D_{i}v\right\|}_{2}+\alpha \left\|v-u\right\|=\underset{v}{\min}\underset{p\subset X}{\max}\langle p,Dv\rangle +\lambda {\left\|v-u\right\|}_{2}^{2}. \end{array}$$

As *X*={*p*=(*p*_1_ ⋯ *p*_*N*_)} ∈ *C*^2*N*^, the primal and dual variables were iteratively updated through the PDHG algorithm, obtaining the following equation:

(12) $$\begin{array}{c} p^{k+1}=\arg\underset{p}{\max}\emptyset \left(v^{k},p\right)+\frac{1}{2\tau_{k}}{\left\|p-p^{k}\right\|}_{2}^{2},\\ v^{k+1}=\arg\underset{v}{\min}\emptyset \left(v,p^{k+1}\right)+\frac{1}{2\theta_{k}}. \end{array}$$

In this equation, ∅(*v*^*k*^, *p*)=〈*p*, *Dv*〉+*α*‖*v* − *u*^*k*^‖_2_^2^; and *τ*_*k*_ and *θ*_*k*_ represented the primal and dual step-size, respectively, corresponding to the total variational regularization term. The final iteration result was expressed as (13)

(13) $$\begin{array}{c} p^{k+1}={\prod^{\;}}_{X}\left(p^{k}+\tau_{k}Dv^{k}\right),{\left({\prod^{\;}}_{X}\left(p\right)\right)}_{i}=\frac{p_{i}}{\max\left\{{\left\|p_{i}\right\|}_{2},1\right\}},\\ v^{k+1}={\left(1+2\alpha \theta_{k}\right)}^{-1}\left(v^{k}-\theta_{k}D^{T}p^{k+1}+2\alpha \theta_{k}u^{k}\right)\left(1-\theta_{k}\right)v^{k}+\theta_{k}\left(u^{k}-\frac{1}{2\alpha }D^{T}p^{k+1}\right). \end{array}$$

For the second sub-issue in (10), dual decomposition method could be used to solve them .

(14) $$\begin{array}{c} \underset{u}{\min}\lambda {\left\|\text{Au}-f\right\|}_{2}^{2}+\alpha {\left\|v-u\right\|}_{2}^{2}. \end{array}$$

Then, the equation of the second sub-issue was obtained, which was expressed as follows:

(15) $$\begin{array}{c} u^{k+1}=\arg\underset{u}{\min}\lambda \left(2{\left(Au^{k}-f\right)}^{T}A\left(u-u^{k}\right)+\delta_{k}{\left\|u-u^{k}\right\|}_{2}^{2}+\alpha {\left\|v-u\right\|}_{2}^{2}\right). \end{array}$$

In the equation, *δ*_*k*_=‖*A*(*u*^*k*^ − *u*^*k*−1^)‖_2_^2^/‖*u*^*k*^ − *u*^*k*−1^‖_2_^2^. The matrix *A* had the very complicated and ill-conditioned structure in parallel MRI, in which under the total variational model the *S*_*j*_*u* in (3) could be replaced by *X*_*j*_ for variable decomposition. Then, (3) was transformed into the following:

(16) $$\begin{array}{c} \underset{u\in C^{N}}{\min}{\left\|u\right\|}_{TV}+\lambda \overset{K}{\underset{j=1}{\sum }}{\left\|{\text{MFX}}_{j}-f_{j}\right\|}_{2}^{2},X_{j}=S_{j}u. \end{array}$$

With the augmented Lagrangian function, (16) could then be transformed into (17).

(17) $$\begin{array}{c} {\left\|u\right\|}_{TV}+\lambda \overset{K}{\underset{j=1}{\sum }}{\left\|MFX_{j}-f_{j}\right\|}_{2}^{2}+2\alpha \langle d_{j},X_{j}-S_{j}u\rangle +\alpha {\left\|X_{j}-S_{j}u\right\|}_{2}^{2}. \end{array}$$

Then, (17) was iterated with the alternating direction multiplier method, and (18) was worked out.

(18) $$\begin{array}{c} X_{j}^{k+1}=\arg\underset{X_{j}}{\min}{\left\|{\text{MFX}}_{j}-f_{j}\right\|}_{2}^{2}+2\alpha \langle b_{j}^{k},X_{j}-S_{j}u^{k}\rangle +\alpha {\left\|X_{j}-S_{j}u\right\|}_{2}^{2},\\ u^{k+1}=\arg\underset{u}{\min}{\left\|u\right\|}_{TV}+\lambda \alpha \langle b_{j}^{k},X_{j}-S_{j}u^{k}\rangle +\alpha {\left\|X_{j}-S_{j}u\right\|}_{2}^{2},\\ b_{j}^{k+1}=b^{k}+\left(X_{j}^{k+1}-S_{j}u^{k+1}\right).j=1\cdots N. \end{array}$$

Since the solution of the matrix in the conventional equation was the product of the Fourier transform and the diagonal matrix, *F*^*T*^*M*^*T*^*MF*+*αI*=*F*^*T*^(*M*^*T*^*M*+*αI*)*F* in the (18). The *u-*sub-issues of X_*j*_^*k*+1^ in the (18) could be quickly calculated through the PDHG algorithm.

### 2.4. Image Evaluation Indicators

The peak signal-to-noise ratio (PSNR) could evaluate the quality of the processed images, which is commonly used in the fields of super-resolution, compression, and restoration of images. PSNR was expressed as the logarithm of the ratio of the mean square error (MSE) between the reconstructed image and the true image to the maximum possible pixel value of the image [17].

The MSE was a measure of the error between the reconstructed image and the true value image, and its specific definition was expressed as follows:

(19) $$\begin{array}{c} \text{MSE}=\frac{1}{H\times W}\overset{H}{\underset{i=1}{\sum }}\overset{W}{\underset{j=1}{\sum }}{\left(f\left(i,j\right)-g\left(i,j\right)\right)}^{2}. \end{array}$$

In this equation, *f(i,j)* and *g(i,j)* represented the reconstructed image and the true value image, respectively, with the height *H* and the width *W*. The PSNR of these two images could be calculated through the MSE and the maximum possible pixel value. PSNR was defined as the following:

(20) $$\begin{array}{c} \text{PSNR}=10\;\log_{10}\left(\frac{{\left(2^{n}-1\right)}^{2}}{\text{MSE}}\right). \end{array}$$

In equation (20), *n* represented the number of bits of the image pixel, and *2*^*n*^*-1* represented the maximum possible pixel value of the image. The unit of PSNR was dB; and the larger the PSNR, the better the reconstruction quality of the image.

Structural similarity image measurement (SSIM) consisted of three contrasts of the brightness, contrast, and structure. The calculation equation of this indicator is shown as (21) below:

(21) $$\begin{array}{c} \text{SSIM}\left(x,y\right)=\frac{\left(2\mu_{x}\mu_{y}+C_{1}\right)\left(2\sigma_{xy}+C_{2}\right)}{\left(\mu_{x}^{2}+\mu_{y+}^{2}C_{1}\right)\left(\sigma_{x}^{2}+\sigma_{y+}^{2}C_{2}\right)}. \end{array}$$

### 2.5. Multimodal MRI Examination Methods

Before the examination, all metal objects, such as bracelets and intrauterine devices, were removed. The bladder was filled appropriately to avoid fluctuation artifacts caused by excessive urine. The patients were in a supine position with hands raised above the head, and the head would enter the device range at first. The patients were comforted to stay relaxed, were trained to hold their breath or breath naturally, and an indwelling needle on the elbow was given to enhance the scanning of injection angiography.

A 1.5 T MRI instrument was used with a body-phased array surface coil. The scanning range was about from the horizontal line of the umbilical cord to the pelvic floor. The conventional plain scanning was carried out in order of T1, T2, diffusion weighted imaging (DWI), and dynamic contrast enhanced MRI (DCE-MRI). Conventional scanning in the coronal, sagittal, and axial positions were mainly included.

### 2.6. Statistical Methods

SPSS22.0 was used to process the relevant data. The medical examination results were taken as the gold standards. The coincidence and Kappa value of the two examination methods were compared, and their values in the diagnosis and staging of early cervical cancer were evaluated. The enumeration data were described by rate (%); and the measurement data, conformed to the normal distribution and the homoscedasticity, were compared under the paired *t*-test, and *P* < 0.05 was considered to be statistically significant.

## 3. Research Results

### 3.1. Result Analysis of Image Reconstruction Indicators

**Figure 1:**
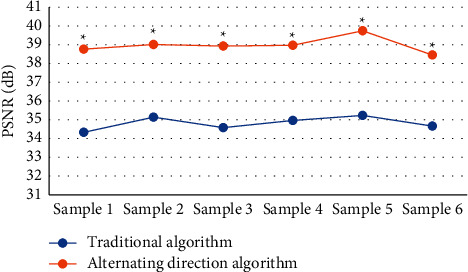
Comparison of PSNR under different image processing methods.  ^*∗*^indicates that compared with the traditional algorithm, the differences were statistically significant as *P* < 0.05.

**Figure 2:**
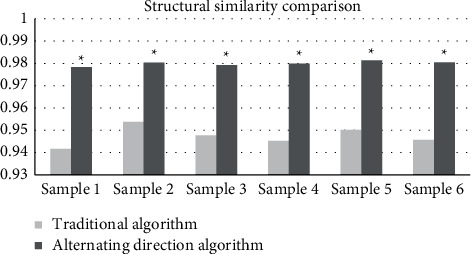
Comparison of SSIM under different image processing methods.  ^*∗*^indicates that the differences were statistically significant compared with those of the traditional algorithm, *P* < 0.05.

The imaging effect of multimodal MRI based on the alternating direction algorithm was compared with that of conventional multimodal MRI. The results showed that for the 6 randomly selected samples of early cervical cancer, the average PSNR and the average SSIM of the traditional algorithm was 34.82 dB and 0.9474, respectively. The average PSNR of the alternating direction algorithm was 38.98 dB, and the average SSIM was 0.9799. The MRI under the alternating direction algorithm had significant advantages in the image reconstruction. The PSNR and SSIM of the algorithm-based MRI were significantly higher than those of conventional MRI, with the reconstruction effect was significantly improved. The comparisons are shown in Figures 1 and 2.

**Figure 3:**
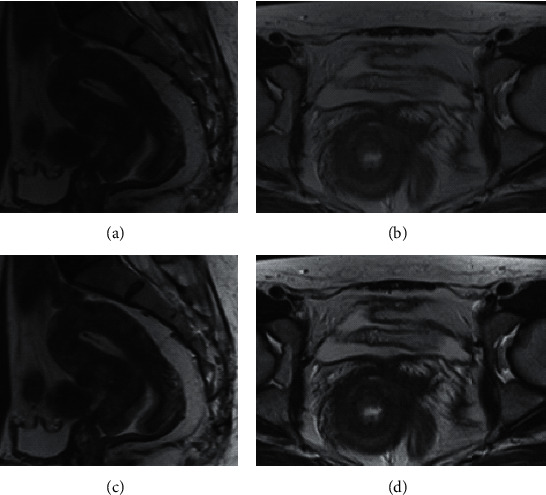
Imaging comparison of two algorithms. A and C are the images of conventional multimodal MRI; B and C are those of multimodal MRI under the alternating direction algorithm.

As shown in Figure 3, the multimodal MRI images of the same patient with early cervical cancer were compared, between those of conventional MRI and those of MRI under the alternating direction algorithm. It can be observed that the multimodal MRI under the alternating direction algorithm gave the significantly clearer images than the conventional multimodal MRI in the corresponding level (the arrows pointed to the tumors).

### 3.2. Comparison of Multimodal MRI and Medical Examination

**Figure 4:**
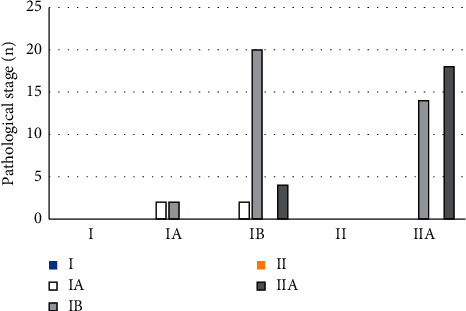
Result comparison of conventional MRI and medical examination.

As shown in Figure 4, the results of conventional multimodal MRI showed that there were missed diagnoses in 2 cases, and the sensitivity reached 96.88% (62/64). With the postoperative pathological staging, it was suggested that the examination results of 38 of the 64 cases with early cervical cancer were consistent with the postoperative pathological staging results, and the staging accuracy was 59.38% (38/64).

**Figure 5:**
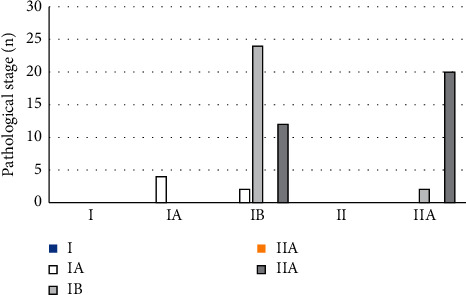
Result comparison of MRI under alternating direction algorithm and medical examination.

The multimodal MRI examination under the alternating direction algorithm showed that there was no missed diagnosis, and the sensitivity reached 100% (64/64). As shown in Figure 5, the MRI examination results of 48 of the 64 cases with early cervical cancer were consistent with those of postoperative pathological staging, and the staging accuracy was 75% (48/64).

### 3.3. Consistency between the Staging Obtained by the Two Methods and Postoperative Medical Examination

**Figure 6:**
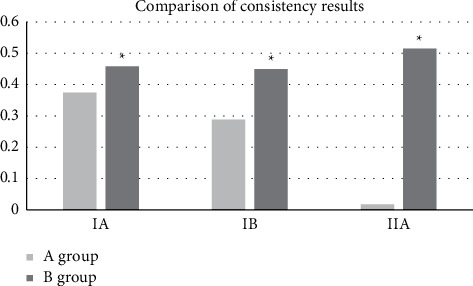
Consistency comparison among the two methods and medical examination.  ^*∗*^indicates that compared with the Kappa coefficient of A group, the differences were statistically significant (*P* < 0.05).

Generally speaking, the Kappa coefficient ranged between [−1,1]. When the Kappa coefficient was 0.4–0.75, it meant that the results were highly consistent; when the Kappa coefficient was ≤0.4, the consistency was relatively worse. According to the postoperative pathological results, the early cervical cancers in patients were staged at I-A, I-B, and II-A, respectively. The weighted Kappa coefficients of conventional MRI were 0.3748, 0.2885, and 0.0173, respectively, which were all less than 0.4, indicating that the consistency between them and the pathological results was poor. The Kappa coefficients of algorithm-based MRI were 0.4583, 0.4497, and 0.5152, respectively, which were all greater than 0.4. Thus, as shown in Figure 6, the staging of multimodal MRI under the alternating direction algorithm was significantly superior to that of conventional multimodal MRI in the diagnosis of early cervical cancer, with the statistically significant difference (*P* < 0.05).

### 3.4. Evaluation of the Diagnosis Results with the Two Examination Methods

**Figure 7:**
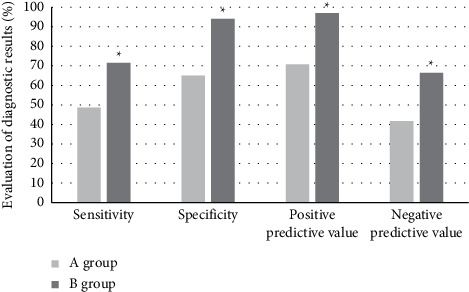
Evaluation of the diagnosis results with the two examination methods.  ^*∗*^indicates that the differences were statistically significant, as the data were compared with those of A group (*P* < 0.05).

The postoperative pathological results were taken as the gold standard. The sensitivity, specificity, positive predictive value, and negative predictive value of the two examination methods were calculated for the diagnosis of early cervical cancer. It is shown in Figure 7 with the results that the values of the four items of group A were 48.7, 65.1, 70.7, and 41.8, respectively; while those of group B were 71.5, 94, 97, and 66.4, respectively.

**Figure 8:**
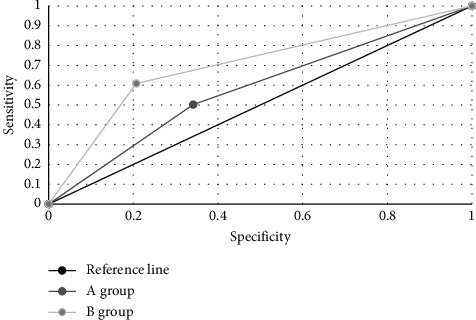
ROC curves of the two examination methods.

The two examination methods were drawn into the receiver operating characteristic (ROC) curves, as shown in Figure 8. The area under the ROC curve (AUC) was 0.617 of conventional multimodal MRI, and the standard error (SE) was 0.7, the 95% confidence interval (CI) of the area was (0.408, 0.716), the sensitivity was 0.503, and the misdiagnosis rate was 0.349. For the multimodal MRI under the alternating direction algorithm, the AUC, SE, the 95% CI, the sensitivity, and the misdiagnosis rate was 0.752, 0.048, (0.766, 0.957), 0.614, and 0.133, respectively. It could be observed that the AUC of the algorithm-based MRI in this study was larger than that of the conventional multimodal MRI, the sensitivity was significantly improved, and the misdiagnosis rate was significantly reduced.

## 4. Discussion

Cervical cancer is the gynecological malignant tumor with the highest incidence. The morbidity and mortality of it remain high in developing countries, and the clinical staging determines the final treatment measures. The clinical staging of cervical cancer mainly relies on pelvic examination currently, and the diagnosis is also highly subjective. Together with the unreliable staging, it often makes patients miss the optimal treatment plan, leading to a poor prognosis [18]. With the medical development, MRI examination of cervical cancer is becoming more and more important for the diagnosis and staging of cervical cancer. The accurate staging depends on clearly showing of the anatomical structure of the various organs in the pelvic cavity, the levels among the various tissues, and the difference between the tumor and normal tissues [19]. Multimodal MRI with multidimensional and multiparameter imaging shows the anatomical relationship very clearly among various organs and tissue structures in the pelvic cavity. The high soft tissue resolution provides an intuitive anatomical basis for the judgment of cervical cancer invasion and the depth of infiltration. The clear structures shown make the preoperative evaluation of cervical cancer more accurate, but there are still many disadvantages [20]. Therefore, in this study, the MRI based on the alternating direction algorithm was applied for the examination of patients. The results of the algorithm-based MRI were compared with those of conventional MRI and clinical examination, to explore its application effect in the diagnosis of early cervical cancer.

It was shown that among the 6 randomly selected samples of early cervical cancer patients, the multimodal MRI under the alternating direction algorithm had a significant advantage in image reconstruction. For the traditional algorithm, the average values of PSNR and SSIM were 34.82 dB and 0.9474, respectively. For the alternating direction algorithm, the average PSNR and the average SSIM were 38.98 dB and 0.9799, respectively. The values were also significantly higher than those of conventional multimodal MRI. With the algorithm-based MRI, the reconstruction effect was significantly improved, and the reconstructed images were clearer. The multimodal MRI under the alternating direction algorithm was used to assist in the staging of early cervical cancer and then was compared with the staging of conventional multimodal MRI. The staging accuracy of B group was 75%, while that of A group was only 59.38%. For the postoperative medical examinations, it was shown from the postoperative staging results that the weighted Kappa coefficient of conventional multimodal MRI for cervical cancer stage IA, IB, and IIA were 0.3748, 0.2885, and 0.0173, respectively. The Kappa coefficients of multimodal MRI under the alternating direction algorithm were 0.4583, 0.4497, and 0.5152, respectively. The consistency with B group was far better than that with the A group, and the difference was statistically significant (*P* < 0.05), which proved that the algorithm-based MRI was more accurate in the staging of early cervical cancer than the conventional MRI. In addition, the AUC of conventional multimodal MRI was 0.617, the standard error was 0.7, the 95% confidence interval of the area was (0.408, 0.716), the sensitivity was 0.503, and the misdiagnosis rate was 0.349. For the multimodal MRI under alternating direction algorithm, the AUC, standard error, 95% confidence interval of the area, sensitivity, and the misdiagnosis rate was 0.752, 0.048, (0.766, 0.957), 0.614, and 0.133, respectively. The AUC of the algorithm-based MRI was larger than that of the conventional multimodal MRI, showing that the sensitivity of the algorithm-based MRI was significantly improved, and the misdiagnosis rate was significantly reduced. Therefore, it was more valuable for the diagnosis of early cervical cancer.

All in all, the multimodal MRI under the alternating direction algorithm was of great value in the diagnosis and staging of early cervical cancer. It was easier to find small lesions with the algorithm-based MRI, thereby the detection rate and staging accuracy of the lesions were improved. Thus, it could be taken as one of the routine examination methods for patients with cervical cancer.

## 5. Conclusion

Multimodal MRI under the alternating direction algorithm was superior to conventional multimodal MRI in the diagnosis of early cervical cancer. As the lesions were shown more clearly, the detection rate and staging accuracy of small lesions were improved. Therefore, it could be used as an ideal MRI method to assist in the staging of cervical cancer with the application value. Although this algorithm-based MRI made some improvement in the accuracy of examination, it still cannot achieve the effect of 100% accuracy of cervical cancer staging. In the follow-up researches, this technology needed to be further improved and developed.

## Figures and Tables

**Table 1:** Cervical cancer staging standards.

FIGO staging		Tumor node metastasis (TNM)
	The carcinoma in situ was unassessable.	TX
	No evidence of primary cancer was found.	T0
Stage 0	Carcinoma in situ (pre-invasive carcinoma) was found.	Tisn0m0
Stage I	Cervical cancer was confined to the uterus (that spread to the uterus was not considered in staging.).	T1N0M0
I-A	Invasive carcinoma was found under a microscope, including superficial infiltration and any visible lesions.	T1a
I-A_1_	Interstitial infiltration depth was <3 mm, and horizontal diffusion range was ≤7 mm.	T1a_1_
I-A_2_	Interstitial infiltration depth reached 3–5 mm, and horizontal diffusion range was ≤7 mm.	T1a_2_
I-B	The microscopic lesion was larger than that of stage I-A_2_, or visible cancer lesions were confined to the cervix.	T1b
I-B_1_	The maximum diameter of the macroscopic cancer lesions was ≤4 cm.	T1b_1_
I-B_2_	The maximum diameter of the macroscopic cancer lesions was >4 cm.	T1b_2_
Stage II	The cancer spread out of the uterus, but did not reach the pelvic wall or the lower third of the vagina.	T2N0M0
II-A	No parauterine infiltration was found.	T2a
II-B	Parauterine infiltration could be observed.	T2b

## Data Availability

The data used to support the findings of this study are available from the corresponding author upon request.
